# Understanding the gender disparity in HIV infection across countries in sub-Saharan Africa: evidence from the Demographic and Health Surveys

**DOI:** 10.1111/j.1467-9566.2010.01304.x

**Published:** 2011-05-04

**Authors:** Monica Akinyi Magadi

**Affiliations:** Department of Sociology, City University London

**Keywords:** gender disparity, HIV infection, sub-Saharan Africa, cross-national variation, Demographic and Health Surveys

## Abstract

Women in sub-Saharan Africa bear a disproportionate burden of human immunodeficiency virus (HIV) infections, which is exacerbated by their role in society and biological vulnerability. The specific objectives of this article are to (i) determine the extent of gender disparity in HIV infection; (ii) examine the role of HIV/acquired immune deficiency syndrome (AIDS) awareness and sexual behaviour factors on the gender disparity and (iii) establish how the gender disparity varies between individuals of different characteristics and across countries. The analysis involves multilevel logistic regression analysis applied to pooled Demographic and Health Surveys data from 20 countries in sub-Saharan Africa conducted during 2003–2008. The findings suggest that women in sub-Saharan Africa have on average a 60% higher risk of HIV infection than their male counterparts. The risk for women is 70% higher than their male counterparts of similar sexual behaviour, suggesting that the observed gender disparity cannot be attributed to sexual behaviour. The results suggest that the risk of HIV infection among women (compared to men) across countries in sub-Saharan Africa is further aggravated among those who are younger, in female-headed households, not in stable unions or marital partnerships or had an earlier sexual debut.

## Introduction

### Background

Even though there is evidence that the overall human immunodeficiency virus (HIV) prevalence in most of sub-Saharan Africa has stabilised or started to decline, the actual number of people infected continues to grow because of ongoing new infections and increasing access to anti-retroviral therapy which extends the lives of those living with AIDS (UNAIDS 2008, UNAIDS and the World Health Organization WHO [2009]). Women bear a disproportionate burden of HIV infections in the region, accounting for nearly 60% of HIV infections (UNAIDS 2008). Since 1985, when it was estimated that roughly equal numbers of women and men in sub-Saharan Africa were living with HIV/AIDS, the number of women living with HIV/AIDS relative to men has increased every year, particularly among the 15–24-year olds (UNAIDS 2004: 2).

There are important differences between women and men in the underlying mechanisms of HIV infection and in the social and economic consequences of HIV/AIDS, which result from biology, sexual behaviour and socially constructed gender differences between men and women in roles and responsibilities, access to resources and decision-making power (Madkan *et al.* 2006, WHO 2003). In a study of 730 stable couples in 16 Italian clinic centres, Nicolosi *et al.* (1994) found that the efficiency of male-to-female transmission was 2.3 times greater than that of female-to-male transmission. Existing studies in other settings confirm that it is much easier for a woman to contract HIV from sexual contact with a man than it is for a man from a woman^1^ and suggest that pregnancy-related complications such as haemorrhage further expose women to risk of infection related to blood transfusion (WHO 2003). These factors are particularly important in sub-Saharan Africa where heterosexual sex is the main mode of HIV transmission and fertility levels in a number of countries in the region have remained particularly high.

The gender disparity in HIV infection is particularly pronounced among young people. In some parts of sub-Saharan Africa the prevalence of HIV infection among women aged 15–24 is two to eight times that among men in the same age group (Clark *et al.* 2006, Madkan *et al.* 2006, UNAIDS 2004). For instance, women aged 15–24 years in nine southern Africa countries most affected by HIV were on average about three times more likely to be infected than men of the same age (Gouws *et al.* 2008). In a study in Kisumu (Kenya) and Ndola (Zambia), Glynn *et al.* (2001) observed that in both sites HIV prevalence in women was six times that in men among sexually active 15–19-year olds; three times that in men among 20–24-year olds and equal to that in men among 25–49-year olds. Such disparities have been partly attributed to biological and psychosocial factors (Rosenthal *et al.* 1999, UNAIDS 2003), and partly to women having older sexual partners who have more sexual exposure (MacPhail *et al.* 2002, Sa and Larsen 2008, WHO 2003), especially in societies where social norms dictate marriage at an early age for women. Research from 16 countries in sub-Saharan Africa indicates that husbands of 15–19 year old girls are on average 10 years older than their wives (WHO 2003).

Other factors contributing to increased vulnerability of women to HIV infection (and other sexually transmitted diseases) in sub-Saharan Africa include gender-based discrimination, violence and economic vulnerability (Ackermann and De Klerk 2002, Dunkle *et al.* 2004, Türmen 2003, Upchurch and Kusunoki 2004). Existing gender differences in power relations and decision-making in most of sub-Saharan Africa may lead to women experiencing violence when attempting to negotiate safer sex or being coerced into sex, increasing their risk of HIV infection.^2^ Women are particularly at risk of rape or sexual assault in conflict situations. As noted by the World Health Organization (WHO 2003), conflict situations exacerbate a number of factors that fuel the HIV/AIDS crisis, including breakdown of families and communities, forced displacement, poverty, the collapse of health services and physical and sexual violence.

Women’s vulnerability to HIV infection is further aggravated by their inability to negotiate safe sex through condom use. A study in Botswana and South Africa noted that the gender power imbalance significantly affected women’s ability to suggest condom use to their partners (Langen 2005). In particular, older male partners (that is, 10 years older) abused women, and women economically dependent on their partners were observed to be less likely to suggest condom use to their partners. Although use of female condoms can substantially reduce the risk of acquiring HIV the price of the female condom (4–10 times that of male condom) makes it inaccessible to most women (WHO 2003).

In general, the basic background patterns of gender disparities in HIV infection (that is, the disproportionate higher risk among women than men in younger age groups) observed from previous studies are generally consistent, but the patterns with respect to risky behaviour or to some indicators of gender inequality remain inconclusive. For example, the higher risk of HIV infection among women than men persists, despite men exhibiting riskier behaviour such as having multiple sex partners (Boileau *et al.* 2009) or their lower use of HIV testing services (Obermeyer *et al.* 2009). Also, an earlier sexual debut has been observed to be risk-inducing in women yet risk-reducing in men (Boileau *et al.* 2009). Furthermore Jewkes *et al.* (2003) found both positive and negative associations between some indicators of gender inequality, HIV and condom use, and therefore highlighted the need for a more nuanced understanding of gender inequalities and their relationship to HIV risk. Existing studies have addressed various aspects of gender disparity in HIV infection in specific settings of sub-Saharan Africa but the overall picture across the sub-Saharan Africa region as a whole is not yet well understood.

### Study objectives

This article aims to contribute to our understanding of the general patterns of gender disparity in HIV infection across countries in sub-Saharan Africa. The main focus is on the extent of gender disparity in HIV infection and the role of individual socioeconomic, HIV/AIDS awareness and sexual behaviour factors in explaining the gender disparity. The specific objectives are to

- determine the extent of gender disparity in HIV infection across countries in sub-Saharan Africa
- examine the role of HIV/AIDS awareness and sexual behaviour factors on the gender disparity
- establish how the gender disparity in HIV infection varies between individuals of different characteristics and across countries in sub-Saharan Africa.

Throughout the analysis, emphasis is placed on cross-national variations and the extent to which the observed gender disparity varies across individuals of different characteristics.

## Data and methods

### The data

This article is based on secondary analysis of existing data from the international Demographic and Health Surveys (DHS) programme from different countries in sub-Saharan Africa (SSA). It uses recent DHS data collected from 20 countries in SSA during the mid-2000s to explore the gender disparity in HIV infection. The comparative nature of DHS data, along with the availability of HIV test data from recent surveys, provides a unique opportunity for a population-based study of factors associated with the HIV/AIDS epidemic in different contexts.

Since 2001 the DHS Programme has conducted nationally representative population-based HIV testing in the DHS or AIDS Indicator Surveys (AIS) in a number of developing countries. This article is based on data collected by the DHS programme during the period 2003–2008 from a total of 20 countries in sub-Saharan Africa. A summary of DHS surveys in SSA analysed in this article is given in Table 1. The linkage of DHS HIV test results to the full DHS survey record (without personal identifiers) allows for an in-depth analysis of the socio-demographic and behavioral factors associated with HIV infection.

**Table 1:** Summary of Demographic and Health (and AIDS Indicator) surveys analysed in the study

	Women		Men	
		
Country	Cases	% HIV+	Cases	% HIV+
Burkina Faso 2003	4189	1.8	3341	2.0
Cameroon 2004	5154	6.6	5041	3.9
Cote d’Ivoire 2005^1^	4535	6.4	3893	2.9
DR Congo 2007	4632	1.6	4304	0.9
Ethiopia 2005	5942	1.9	5107	0.9
Ghana 2003	5289	2.7	4265	1.6
Guinea 2005	3842	1.9	2925	1.1
Kenya 2003	3271	8.7	2917	4.6
Liberia 2007	6482	1.9	5190	1.2
Lesotho 2004–05	3020	26.4	2232	18.9
Malawi 2004	2864	13.3	2404	10.2
Mali 2006	4743	1.5	3886	1.1
Niger 2006	4441	0.7	3232	0.7
Rwanda 2005	5663	3.6	4728	2.2
Senegal 2005	4466	0.9	3250	0.4
Sierra Leone 2008	3466	1.7	3009	1.2
Swaziland 2006	4584	31.1	3602	19.7
Tanzania 2003–2004^1^	5969	7.7	4774	6.3
Zambia 2007	5713	16.1	5161	12.3
Zimbabwe 2005–2006	7494	21.1	5555	14.7
All (sub-Saharan Africa)	95759		78833	

1 AIDS Indicator Survey.

The DHS or AIS HIV testing protocol^3^ provides for the informed, anonymous and voluntary testing of women and men interviewed. Since the testing is anonymous, survey respondents cannot be provided with their results. However, all respondents are offered referrals for free voluntary counselling and testing (VCT) and AIDS educational materials. In some countries mobile VCT teams follow up after interviewers to counsel and test willing DHS respondents.

By collecting blood for HIV testing from representative samples of the population of men and women in a country, the DHS can provide nationally representative estimates of HIV rates. However, it is important to recognise that population-based testing is dependent on the population’s willingness to be voluntarily tested for HIV. Where the characteristics of those who agreed to be tested are different from those who refused testing, bias may result. Coverage for HIV testing is given in Table A1 in the Appendix and a discussion of potential implications of possible non-response bias on the results included in the final section. The overall coverage is reasonable, ranging from a low of 70% for women in Malawi and 63% for men in Malawi and Zimbabwe, to a high of 97% and 95% for women and men, respectively in Rwanda.

### Methods of analysis

The analytical strategy starts with a descriptive analysis of the gender disparity in HIV awareness and of the gender disparity by basic demographic factors, before focusing on multivariate analysis of the extent of gender disparity in HIV infection in individual countries and across countries in sub-Saharan Africa. The descriptive analyses, based on bivariate distributions, are useful for insightful interpretation of findings from the multivariate analysis.

The outcome variable of interest in the multivariate analysis is HIV infection while the key explanatory variable is sex of the respondent, which is used as a proxy for gender (see Table A2 in the Appendix for a description of all covariates). Other explanatory variables considered as covariates in the analysis include

- background individual/household socioeconomic and demographic characteristics (for example, age, urban/rural residence, education, sex of household head, religion, household socioeconomic status)
- HIV/AIDS awareness (that is, general awareness, modes of HIV transmission, ways of preventing infection)
- Sexual behaviour factors (marital union status, age at first marriage, age at first sex, premarital sex, condom use, non-spousal sex partner, multiple partners).

These covariates are introduced to the models in successive stages to establish their role in moderating the gender disparity, starting with background socioeconomic and demographic characteristics, before introducing the HIV/AIDS awareness and finally sexual behaviour factors. We postulate that the covariates included in the model can help explain the gender disparity. For instance, one might expect that women have lower HIV/AIDS awareness than men and therefore are less likely to adopt safer sexual behaviour that would reduce their chances of getting infected with the HIV virus. We further explored interactions of various covariates with sex to establish possible variations of the gender disparity by various individual characteristics.

The multivariate analysis is based on multilevel modelling, applied to pooled DHS and AIS data across countries. Pooling the data across countries is feasible since the DHS and AIS surveys are designed to collect cross-nationally comparable data. The multilevel methodology takes into account the hierarchical structure of the data resulting from pooling data across countries. Furthermore, the methodology is appropriate for this study as it allows for the inclusion of random effects to enable us to control for unobserved characteristics at region and country levels. The analysis places particular emphasis on country and regional variations in factors associated with HIV/AIDS, and the extent of variation in the gender disparity across countries, and across regions within countries. The general form of the multilevel logistic regression model^4^ used may be expressed as:


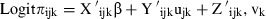
(1)

where *π*_ijk_ is the probability of HIV infection for a specific individual *i*, in the *j*^ *th*^ region in the *k*^*th*^ country; *X’*_*ijk*_ is the vector of covariates that may be defined at the individual/household, community or country level; β is the associated vector of usual regression parameter estimates; *Y’*_*ijk*_ is a vector of covariates (usually a subset of *X’*_*ijk*_) which vary randomly at region level; *Z’*_*ijk*_ is a vector of covariates (usually a subset of *X’*_*ijk*_) which vary randomly at country level; and the quantities *v*_*k*_*,*and *u*_*jk*_ are the residuals at the country and region level, respectively. These are assumed to have normal distribution with mean zero and variances *σ*^*2*^_*v*_ and *σ*^*2*^_*u*_ (Goldstein 2003). In particular, the multilevel models allowed the gender effect to vary randomly at community (region) and country levels.

## Results

### Descriptive analysis

#### Gender disparity in HIV/AIDS awareness

We start by examining gender disparities in HIV/AIDS awareness since this may have important implications for HIV infection. Those with better HIV/AIDS awareness are likely to adopt appropriate behaviour to minimise their risk of infection. In the DHS respondents were asked about their knowledge of ways of avoiding HIV infection as well as awareness of modes of transmission. A set of eight questions (see Table A3 in the Appendix) were used to construct an awareness index, through principal components analysis, and the resulting awareness score classified into tertiles. The tertiles divide the population into three equal subgroups such that the first tertile (low) represents the 33% of the respondents with the lowest awareness, while the highest tertile (high) represents the top 33% with the highest awareness. It is important to recognise that the relationship between HIV awareness and HIV infection is not a straightforward one due to possible endogeneity and it should be interpreted with caution. While it is possible that people who are more aware would be less likely to become infected with HIV, it is also possible that those who develop HIV/AIDS symptoms and then discover they are HIV positive become more aware about HIV as a consequence*.*

Table 2 gives the percentage of women in each awareness tertile by country. A higher proportion of women in the lower awareness tertiles than in the overall sample suggest that women have lower awareness than men, while a higher proportion of women in the higher tertiles suggest the converse.

**Table 2:** Percent of women by HIV/AIDS awareness tertile

	HIV/AIDS awareness				
			
Country	Low	Mid	High	All	Gender sig.
Burkina Faso 2003	64.7	47.4	51.5	54.4	***
Cameroon 2004	60.9	45.8	43.9	50.3	***
Cote d’Ivoire 2005	58.4	51.7	45.3	52.3	***
Democratic Rep. Congo 2007	54.7	47.8	48.0	50.3	***
Ethiopia 2005	65.6	50.0	40.9	52.0	***
Ghana 2003	60.1	52.4	47.5	53.3	***
Guinea 2005	70.0	53.9	42.9	55.3	***
Kenya 2003	56.3	49.2	47.5	50.9	***
Lesotho 2004–2005	48.5	57.3	64.0	57.4	***
Liberia 2007	57.6	53.3	50.6	54.4	***
Malawi 2004	52.5	54.2	45.9	51.0	**
Mali 2006	60.0	48.7	49.1	52.5	***
Niger 2006	69.0	55.9	47.0	57.4	***
Rwanda 2005	61.4	48.1	53.5	54.3	***
Senegal 2005	52.7	50.6	56.0	54.7	*
Sierra Leone 2008	67.1	52.3	41.7	53.3	***
Swaziland 2006	47.6	50.7	62.6	54.0	***
Tanzania 2003–2004	63.6	52.5	54.1	53.5	ns
Zambia 2007	48.0	48.6	54.9	50.6	***
Zimbabwe 2005–2006	55.1	52.5	52.3	53.2	**
All (Sub-Saharan Africa)	58.2	50.8	50.1	53.0	***

* *P* < 0.05

** *P* < 0.01

**** *P* < 0.001.

Gender sig.: gender significance; ns: not significant.

In general, a significantly higher proportion of those with low awareness compared to the high awareness are women, suggesting lower HIV/AIDS awareness among women than men. Overall, women make up 58% of those with low awareness, compared to only 50% of those with high awareness. This pattern is consistent in most of the individual countries in sub-Saharan Africa and is particularly strong in specific countries such as Ethiopia, Guinea and Niger. For instance, women in Guinea make up 70% of those with low awareness, yet only 43% of those with high awareness are women. The only exceptions to the general pattern of lower awareness among women are a few countries where there is either no significant gender difference in HIV/AIDS awareness (Tanzania), or the relationship is reversed (Lesotho, Swaziland, Zambia and, to some extent, Senegal).

#### Gender disparity in HIV infection by age group

It is important to examine the gender disparity by age group since the risk of HIV infection varies considerably by age group for both men and women and infection tends to peak at an earlier age for women than men.

The gender disparity is more significant among the younger age groups – HIV prevalence is significantly higher for women than men in most countries among those aged 15–24 years, and in most countries among those aged 25–34 years. Although the gender disparity is not significant among older respondents aged 35 years or older in most countries, in the few countries where it is significant, the prevalence is still higher among women, the only exception being Swaziland.

#### Gender disparity in HIV infection by marital status

Besides age, marital status plays a considerable role in HIV infection and the gender disparity is likely to vary by marital status. We examine in Table 4 the sex differences in HIV infection by marital status.

**Table 4:** Sex differences in HIV infection by marital status

	Percentage HIV positive								
	
	Never married			Currently married			Previously married		
						
Country	women	men	Sig.	women	men	Sig.	women	men	Sig.
Burkina Faso 2003	2.0	0.8	*	1.6	2.8	**	5.5	4.6	ns
Cameroon 2004	3.5	2.1	*	6.2	4.9	*	18.4	6.7	***
Cote d’Ivoire 2005	4.6	1.3	***	6.1	3.6	***	14.9	11.0	ns
Democratic Rep. Congo 2007	0.8	1.1	ns	1.6	0.8	**	3.7	0.9	*
Ethiopia 2005	0.7	0.3	ns	1.6	1.2	ns	6.5	2.4	ns
Ghana 2003	1.1	0.3	**	2.9	2.5	ns	6.3	3.3	ns
Guinea 2005	1.1	0.5	ns	1.6	1.5	ns	8.9	0.0	***
Kenya 2003	4.7	1.5	***	8.0	6.9	ns	23.7	11.1	**
Lesotho 2004	14.9	8.6	***	26.9	29.3	ns	50.8	32.7	***
Liberia 2007	1.6	1.4	ns	1.8	1.1	**	3.1	2.2	ns
Malawi 2004	5.3	1.7	**	12.6	13.9	ns	29.1	16.7	*
Mali 2006	0.4	0.9	ns	1.6	1.1	ns	4.5	1.9	ns
Niger 2006	0.4	0.5	ns	0.5	0.7	ns	5.7	2.8	ns
Rwanda 2005	1.6	1.0	ns	2.8	3.0	ns	12.4	8.3	ns
Senegal 2005	0.3	0.0	*	0.9	0.8	ns	3.8	1.8	ns
Sierra Leone 2008	2.2	1.0	ns	1.4	1.1	ns	4.1	3.1	ns
Swaziland 2006	25.9	9.9	***	32.5	36.4	*	54.1	58.2	ns
Tanzania 2003	3.8	3.0	ns	6.9	7.8	ns	19.7	15.0	*
Zambia 2007	9.1	4.5	***	14.7	15.5	ns	36.9	37.9	ns
Zimbabwe 2005	8.4	4.3	***	20.2	22.7	**	46.3	43.4	ns
All	5.8	4.3	***	6.8	6.9	ns	22.0	15.2	***

* *P* < 0.05

** *P* < 0.01

*** *P* < 0.001.

Sig.: gender significance; ns: not significant.

The previously married category comprises those who are widowed, divorced or separated.

The disproportionate female burden of HIV infection is more noticeable among those who are unmarried, especially the never married. For instance, in Swaziland the sex difference is solely confined to the never married, with never married women having particularly high HIV prevalence (26%) compared to never married men (10%). The patterns observed among those who have never married is likely to be partly attributable to the greater sex difference in HIV prevalence among the younger age groups (see Table 3), since those who are never married are likely to be younger. A particularly strong female disadvantage is observed among the previously married in specific countries, including Cameroon, Guinea and Lesotho. It is only among the currently married where the female disadvantage is not evident, with the relationship not being significant in most countries, and a higher prevalence among women than men observed in about half of the countries where there is a significant sex difference.

**Table 3:** Sex differences in HIV infection by age group

	Percentage HIV positive								
	
	15–24 years			25–34 years			35 + years		
						
Country	women	men	Sig.	women	men	Sig.	women	men	Sig.
Burkina Faso 2003	1.3	0.7	ns	2.5	3.2	ns	2.0	2.5	ns
Cameroon 2004	4.6	1.4	***	10.0	6.4	***	6.4	5.0	ns
Cote d’Ivoire 2005	2.4	0.3	***	10.7	4.1	***	9.0	5.5	**
Democratic Rep. Congo 2007	0.5	1.0	ns	2.3	0.8	**	2.6	0.9	***
Ethiopia 2005	1.1	0.2	***	1.8	1.3	ns	3.0	1.4	**
Ghana 2003	1.2	0.1	***	3.8	1.8	**	3.5	3.1	ns
Guinea 2005	1.2	0.6	ns	2.4	0.9	*	2.2	1.6	ns
Kenya 2003	5.9	1.2	***	12.4	7.0	***	9.0	7.4	ns
Lesotho 2004	15.4	6.0	***	39.8	32.2	**	30.6	27.8	ns
Liberia 2007	1.7	0.6	***	1.9	1.8	ns	2.2	1.5	ns
Malawi 2004	9.1	2.1	***	16.6	14.5	ns	16.4	14.9	ns
Mali 2006	0.9	0.7	ns	2.1	1.5	ns	1.9	1.3	ns
Niger 2006	0.5	0.3	ns	0.9	0.6	ns	0.8	1.1	ns
Rwanda 2005	1.5	0.3	***	4.6	2.8	**	5.9	4.1	*
Senegal 2005	0.4	0.1	*	1.2	0.6	ns	1.3	0.9	ns
Sierra Leone 2008	1.4	0.6	ns	2.3	1.6	ns	1.4	1.3	ns
Swaziland 2006	22.7	5.9	***	47.4	34.3	***	29.7	38.7	***
Tanzania 2003	4.0	3.0	*	10.9	7.6	**	9.6	9.7	ns
Zambia 2007	8.5	4.3	***	22.6	14.3	***	19.5	19.7	ns
Zimbabwe 2005	11.0	4.3	***	31.8	20.3	***	27.1	29.0	ns
All	5.0	1.9	***	11.4	8.0	***	8.9	7.8	***

* *P* < 0.05

** *P* < 0.01

*** *P* < 0.001.

Sig.: gender significance; ns: not significant.

## Multivariate analysis

### Extent of gender disparity in HIV infection in individual countries

Before examining the variations in the gender disparity in HIV across countries in sub-Saharan Africa using multilevel models, we start by running single-level models in each country to examine the extent of gender disparity in individual countries. Part of the main aim of this article is to investigate the role of HIV/AIDS awareness and sexual behaviour on the gender disparity in HIV infection. We examine in Table 5 the sex differences in HIV infection in specific countries, introducing HIV/AIDS awareness and sexual behaviour factors to the model in subsequent stages to establish the role of these proximate factors in the observed sex difference.

**Table 5:** Adjusted odd ratios for HIV infection for women versus men by country

Country	Model 0	Model 1	Model 2	Model 3
Burkina Faso 2003	1.14	1.02	1.00	1.24
Cameroon 2004	1.73*	1.68*	1.68*	1.74*
Cote d’Ivoire 2005	2.40*	2.38*	2.40*	2.60*
Democratic Rep. Congo 2007	1.76*	1.68*	1.68*	1.91*
Ethiopia 2005	1.71*	1.66*	1.74*	1.92*
Ghana 2003	1.68*	1.36	1.38	1.33
Guinea 2005	1.50	1.73*	1.87*	2.94*
Kenya 2003	1.95*	1.79*	1.80*	1.87*
Liberia 2007	1.91*	2.07*	2.13*	2.00*
Lesotho 2004–2005	1.55*	1.50*	1.49*	1.61*
Malawi 2004	1.51*	1.45*	1.46*	1.36
Mali 2006	1.51*	1.58*	1.58*	1.55
Niger 2006	0.85	0.82	0.84	0.83
Rwanda 2005	1.63*	1.54*	1.56*	1.71*
Senegal 2005	2.12*	2.42*	2.44*	1.92
Sierra Leone 2008	1.75*	1.63*	1.63*	1.62
Swaziland 2006	1.88*	1.89*	1.88*	1.74*
Tanzania 2003–2004	1.32*	1.11	1.11	0.96
Zambia 2007	1.37*	1.35*	1.35*	1.43*
Zimbabwe 2005–2006	1.62*	1.63*	1.64*	1.40*

* significant at 5% level (*P* < 0.05).

Model 0 no controls included in the model.

Model 1 controlling for background socioeconomic and demographic factors (i.e. age, urban/rural residence, region, education, sex of household head, household wealth, and religion).

Model 2 controlling for background factors plus HIV/AIDS awareness.

Model 3 controlling for background factors plus HIV/AIDS awareness and sexual behaviour factors (marital status, age at first marriage, age at first sex, premarital sex, non-condom use/non-spousal partner, multiple partners.

The results show that women have a significantly higher risk of HIV infection than men in almost all countries included in the analysis, with the exception of Burkina Faso and Niger. Once the background socioeconomic and demographic factors are controlled for, the sex difference in HIV infection ceases to be significant in Ghana and Tanzania, suggesting that the gender disparity in HIV infection in these two countries may be attributable to these background characteristics. For Guinea, the sex difference is strengthened by controlling for background factors. Controlling for HIV/AIDS awareness and sexual behaviour further strengthens the sex difference, suggesting that if these factors had not been in play, the gender disparities would be greater in the country. In fact, Guinea seems to have the largest sex difference in HIV infection when sexual behaviour, HIV awareness and background factors are controlled for, with women having on average about triple the odds of HIV infection as their male counterparts of similar characteristics. In countries such as Guinea, where controlling for sexual behaviour factors tends to be associated with a general increase in sex difference, this suggests that women in these countries are particularly at a higher risk of HIV infection compared to their male counterparts of similar sexual behaviour. In general, controlling for HIV/AIDS awareness does not significantly alter the sex difference in HIV infection for most countries.

### Gender disparity in HIV infection across countries in sub-Saharan Africa

In this section we examine sex differences in HIV infection across countries in sub-Saharan Africa using three-level logistic regression models applied to pooled DHS data from 20 countries in the region. The results based on odds ratios are presented in Table 6. Regions within countries are considered as the second level while countries constitute the third level. Special consideration is given to variations in the sex difference across countries by allowing the sex effect to vary across countries in random coefficient models.

**Table 6:** Average odds ratios of HIV infection from multilevel logistic regression models (95% confidence intervals are given in square brackets)

Parameter	Model 1	Model 2	Model 3
**Fixed effects**			
Sex of respondent (male)			
female	1.56 [1.44, 1.70]*	1.57 [1.44, 1.71]*	1.68 [1.53, 1.85]*
Age group (40 + )			
15–19	0.15 [0.14, 0.17]*	0.16 [0.14, 0.17]*	0.35 [0.31, 0.39]*
20–29	0.74 [0.70, 0.79]*	0.75 [0.70, 0.79]*	0.94 [0.88, 1.00]
30–39	1.41 [1.33, 1.49]*	1.41 [1.33, 1.49]*	1.49 [1.40, 1.58]*
Residence (urban)			
rural	0.61 [0.57, 0.64]*	0.61 [0.57, 0.64]*	0.63 [0.59, 0.67]*
Education level (none)			
primary	1.26 [1.18, 1.36]*	1.24 [1.16, 1.34]*	1.21 [1.13, 1.31]*
secondary	1.12 [1.04, 1.22]*	1.10 [1.01, 1.19]*	1.12 [1.03, 1.21]*
Sex of household head (male)			
female	1.45 [1.38, 1.52]*	1.44 [1.37, 1.51]*	1.17 [1.11, 1.23]*
Wealth quintile (lowest)			
second	1.16 [1.08, 1.26]*	1.16 [1.07, 1.25]*	1.18 [1.09, 1.28]*
third	1.27 [1.17, 1.37]*	1.26 [1.17, 1.36]*	1.31 [1.21, 1.41]*
fourth	1.46 [1.35, 1.58]*	1.44 [1.33, 1.56]*	1.51 [1.39, 1.63]*
highest	1.26 [1.15, 1.38]*	1.24 [1.13, 1.36]*	1.35 [1.23, 1.48]*
Religion (Catholic/Orthodox)			
Protestant	0.93 [0.87, 0.99]*	0.93 [0.88, 1.00]*	0.95 [0.89, 1.01]
Muslim	0.80 [0.72, 0.89]*	0.81 [0.73, 0.90]*	0.83 [0.75, 0.92]*
HIV/AIDS awareness (low)			
average		1.08 [1.02, 1.14]*	1.06 [1.00, 1.12]
high		1.14 [1.08, 1.21]*	1.08 [1.02, 1.15]*
Marital status (married monogamous)			
married polygamous			1.02 [0.94, 1.10]
previously married			2.51 [2.34, 2.69]*
Age at first marriage (20 + )			
<16 yrs			0.99 [0.89, 1.11]
16–17			0.84 [0.77, 0.92]*
18–19			0.89 [0.83, 0.96]*
Never married			0.98 [0.88, 1.08]
Age at first sex (20 + )			
Never had sex			0.40 [0.35, 0.46]*
<16 yrs			1.08 [1.00, 1.18]
16–17			1.19 [1.10, 1.28]*
18–19			1.12 [1.04, 1.20]*
Premarital sex			1.38 [1.29, 1.48]*
Risky sexual behaviour^1^			1.12 [1.04, 1.20]*
Multiple sex partners			1.33 [1.24, 1.44]*
**Random variances**			
Region constant	0.15 [0.11, 0.19]*	0.15 [0.11, 0.19]*	0.14 [0.10, 0.18]*
Country constant	1.41 [0.51, 2.32]*	1.43 [0.51, 2.34]*	1.44 [0.52, 2.36]*
Country sex	0.02 [0.00, 0.04]*	0.02 [0.00, 0.04]	0.02 [0.00, 0.04]

* Statistical significance at 5% level *P* < 0.05.

1 No condom use at last sex, with non-spousal partner

Model 1 controlling for background socioeconomic and demographic factors.

Model 2 controlling for background factors plus HIV/AIDS awareness.

Model 3 controlling for background factors, HIV/AIDS awareness, and sexual behaviour.

The results in Table 6 show a highly significant sex difference in HIV infection across countries in sub-Saharan Africa. Without controlling for any covariates (not shown), women have on average about 1.60 times the odds of HIV infection as men. The sex difference does not change much when important background socioeconomic and demographic characteristics (that is, age group, educational attainment, urban/rural residence, sex of household head, religion and wealth index – Model 1) or HIV/AIDS awareness (Model 2) are controlled for. However, a notable increase in the average odds ratio is observed when factors relating to sexual behaviour (that is, marital/union status, age at first sex, age at first marriage, premarital sex, risky sexual behaviour and multiple sex partners) are controlled for (Model 3). On average across countries, women have a 68% higher odds of HIV infection than their male counterparts of similar background socioeconomic and sexual behaviour characteristics.

**Figure 1:**
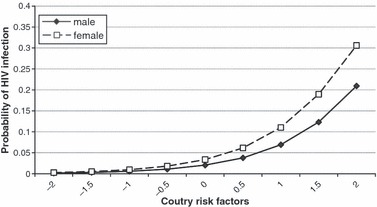
Estimated probabilities of HIV infection for men and women in sub-Saharan Africa at varying country effects, mid 2000s. Note: The estimated probabilities are computed while holding all the other covariates in the model at their mean values

Overall, there are significant variations in the risk of HIV infection across countries in sub-Saharan Africa, and to a lesser extent across regions within countries. These are evident from the significant random variances in Table 6 for the constant (intercept) at country and region levels, and the variances being relatively large at the country level. Hardly any change is noted in the variances after controlling for background characteristics, HIV/AIDS awareness and sexual behaviour factors in Models 1, 2 and 3. Although the gender disparity is fairly consistent across regions within countries (that is, the sex effect at regional level was not significant and hence was excluded from the final model), there is some variation in the gender disparity across countries in sub-Saharan Africa, as suggested by the marginally significant sex effect at country level. Figure 1 shows the variation in the risk of HIV infection for men and women, with varying country effects.^5^

The estimated probabilities in Figure 1 suggest that the sex difference in HIV infection is more pronounced in countries with higher HIV prevalence. After controlling for individual background characteristics and sexual behaviour factors, women in high-risk countries have an average of about 30% risk of HIV infection, compared to a 20% risk for their male counterparts of similar characteristics.

Besides the highly significant sex main effect, the sex differences showed significant variations by a number of factors. The results of further analysis involving interactions of sex with individual characteristics are presented in Table 7 for the significant interactions and corresponding main effects.

**Table 7:** Multilevel logistic regression parameter estimates of HIV infection for significant interactions with sex of respondent

Parameter	Model 1	Model 2	Model 3
**Fixed effects**			
Constant	−3.08 (0.305)	−3.12 (0.307)	−3.57 (0.304)
Sex of respondent (male)			
female	−0.24 (0.109)*	−0.27 (0.114)*	−0.32 (0.094)*
Age group (40 + )			
15–19	−2.44 (0.088)*	−2.44 (0.088)*	−1.66 (0.110)*
20–29	−0.85 (0.050)*	−0.85 (0.050)*	−0.60 (0.056)*
30–39	0.21 (0.044)*	0.21 (0.044)*	0.22 (0.045)*
Age group × sex			
women: 15–19	1.01 (0.104)*	1.02 (0.104)*	0.91 (0.129)*
women: 20–29	0.97 (0.064)*	0.97 (0.064)*	0.91 (0.070)*
women: 30–39	0.30 (0.060)*	0.30 (0.060)*	0.38 (0.062)*
Education level (none)			
primary	0.09 (0.060)	0.08 (0.061)	0.04 (0.060)
secondary	0.05 (0.065)	0.03 (0.065)	0.02 (0.064)
Education level × sex			
women: primary	0.18 (0.072)*	0.18 (0.072)*	0.21 (0.073)*
women: secondary	0.04 (0.078)	0.04 (0.079)	0.11 (0.076)
Sex of household head (male)			
female	0.04 (0.055)	0.04 (0.055)	0.11 (0.057)
Sex of household head × sex			
women: female hhhead	0.50 (0.062)*	0.49 (0.062)*	0.06 (0.65)
Marital status (married)			
never married			−0.34 (0.070)*
married; polygamous			0.09 (0.083)
previously married			0.71 (0.062)*
Marital status *sex			
women: never married			0.70 (0.079)*
women: married; polyg.			0.21 (0.095)*
women: previously married			0.39 (0.072)*
Age at first marriage (20 + )			
<16 yrs			0.32 (0.137)*
16–17			0.03 (0.191)
18–19			0.18 (0.066)*
Age at first marriage × sex			
women: <16 yrs			−0.39 (0.145)*
women: 16–17			−0.22 (0.110)*
women: 18–19			−0.35 (0.079)*
Age at first sex (20 + )			
Never had sex			−0.31 (0.110)*
<16 yrs			0.06 (0.058)
16–17			0.18 (0.054)*
18–19			0.10 (0.051)*
Age at first sex × sex			
female: never had sex			−0.94 (0.140)*
women: <16 yrs			0.20 (0.077)*
women: 16–17			0.10 (0.072)
women: 18–19			0.11 (0.069)
**Random variances**			
Region constant	0.15 (0.022)*	0.15 (0.022)*	0.14 (0.021)*
Country constant	1.57 (0.511)*	1.58 (0.514*	1.59 (0.516)*
Country sex	0.02 (0.012)	0.02 (0.012)	0.03 (0.013)*
Country constant × sex	−0.14 (0.065)*	−0.14 (0.067)*	−0.16 (0.070)*

* Statistical significance at 5% level *P* < 0.05; $ no condom use at last sex, with non-spousal partner.

hhhead, head of household; polyg., polygamous.

Model 1 controlling for urban/rural residence, household wealth and religion, beside shown background factors;

Model 2 controlling for background factors plus HIV/AIDS awareness; and

Model 3 controlling for background factors, HIV/AIDS awareness, and sexual behaviour (including premarital sex, risky sexual behaviour and multiple sex partners, all of which are not shown since interactions with sex are not significant).

The results of the interactions with background factors suggest that the risk of HIV infection among women (compared to men) is further increased among those who are younger, have primary education or live in female-headed households. In fact, the higher risk of HIV infection associated with primary level education or female-headed households observed in Table 6 applies exclusively to women, since the main effects of these factors are not significant when the interactions with sex are included in the model (Table 7). However, the disproportionately higher risk among women in female-headed households vanishes when sexual behaviour factors are controlled for (Model 3), confirming that the higher risk of HIV infection among women in female-headed households is attributable to sexual behaviour.

The sex difference also varies significantly by marital status, age at first marriage and age at first sexual encounter. The higher risk of HIV infection among women is aggravated among those who are unmarried (never married, widowed or divorced/separated) or married in polygamous unions. This is consistent with patterns observed earlier in the descriptive analysis. It is interesting to note that although early marriage tends to be generally associated with an increased risk of HIV infection (as suggested by the somewhat significant and positive main effect), it is associated with a significantly reduced risk of HIV infection among women than among men. Finally, it is important to note that sexual abstinence is more protective for women than men and that the risk of HIV infection is disproportionately higher among women than men who initiate sexual activity before the age of 16 years as opposed to the age of 20 years or older.

## Discussion and conclusions

The main aim of this article was to improve understanding of the gender disparity in HIV infection across countries in sub-Saharan Africa. The specific objectives were to (i) determine the extent of gender disparity in HIV infection across countries in sub-Saharan Africa; (ii) examine the role of HIV/AIDS awareness and sexual behaviour factors on the gender disparity and (iii) establish how the gender disparity in HIV infection varies between individuals of different characteristics and across countries in sub-Saharan Africa.

First, we recognise potential data limitations that should be borne in mind while interpreting our findings. The first relates to the problem of causality since the cross-sectional nature of the data makes it impossible to determine the time sequence of key events of interest, that is, whether the HIV infection preceded various risk factors or whether the observed relationships are due to the effect of predisposing conditions associated with both HIV/AIDS and gender. Hence, the analysis focused on the associations rather than causal relationships. Secondly, we recognise a possible selectivity bias due to the differential coverage in HIV testing by sex of respondent. If HIV-seropositive individuals of a particular sex are relatively more likely to refuse to be tested, then those tested may represent a select low-risk subgroup, distorting the observed gender disparity in HIV infection. The sex differences in HIV testing response rates by country presented in the Appendices (Table A1) suggest generally lower coverage for men than women. However, this is largely due to reasons other than refusal, implying that non-response is more likely to be random than selective. In most countries the main reason for the lower coverage among men than women was because men were more likely to be away from home and less likely to have been interviewed.

Overall, the results suggest that on average the odds of women having an HIV infection were 60% higher than their male counterparts. This does not change when background demographic/socioeconomic characteristics or HIV/AIDS awareness factors are controlled for, suggesting that the risk of infection is on average 60% higher for women than their male counterparts of similar background characteristics and HIV/AIDS awareness. However, compared to their male counterparts of similar sexual behaviour, women have about a 70% higher risk, suggesting that the higher risk of HIV infection among women than men cannot be attributed to sexual behaviour factors. In fact, the patterns suggest that women have a significantly higher risk despite men tending to have riskier sexual behaviour, consistent with patterns observed in previous studies (Boileau *et al*. 2009). The evidence presented here supports the view that behavioural factors cannot fully explain the gender disparity in HIV infection and that the greater biological susceptibility of women to HIV infection is possibly an important factor in explaining the male–female disparity in HIV prevalence (Glynn *et al.* 2001).

The results show significant variations in the gender disparity in HIV infection by various background factors, including age group, educational attainment and gender of household head. The gender disparity by age group is consistent with patterns observed in previous studies (Glynn *et al.* 2001, MacPhail *et al.* 2002), confirming a particularly higher risk among younger women compared to their male counterparts of a similar age. Such disparities have been partly attributed to biological and psychosocial factors (Rosenthal *et al*. 1999, UNAIDS 2003), or to women having older sexual partners who have had greater exposure to the risk of HIV infection (MacPhail *et al*. 2002, Sa and Larsen 2008, World Health Organization (WHO) 2003). The high risk of HIV infection among young women who are still in the early stages of their reproductive period has implications not only for the health of the women but also for vertical transmission of HIV, especially since limiting childbearing may not be a viable option at this stage, even when women are already aware of their HIV status.

This study reveals a particularly strong gender disparity in the risk of HIV infection with respect to the sex of household head. The risk of HIV infection is considerably higher among women in female-headed households compared to their male counterparts in similar households. It is interesting to note that the higher risk of infection among women than men living in female-headed households is almost wholly explained by sexual behaviour. Among older women this may be largely explained by the fact that women in female-headed households are more likely to be widowed, divorced or separated; factors associated with an increased risk of HIV infection. Among younger women the observed patterns might suggest that parental factors are more important for women’s than men’s risky sexual behaviour. Previous studies have noted that parental factors (such as living in the same household as the father) are positively associated with sexual abstinence and a reduced number of sex partners (Babalola *et al.* 2005), and that father’s presence, unlike mother’s, is associated with stronger resilience (that is, less likely to have ever had sex or to be sexually active) among adolescent girls (Ngom *et al.* 2003). This explanation is also supported by further analysis (not shown) which suggests that men in female-headed households are considerably more likely to have never had sex compared to women in similar households. Although it is possible that the unfavourable economic position of women in female-headed households may also be a contributing factor, the fact that the observed patterns persist when household economic circumstances are controlled for may suggest that women’s vulnerability in such circumstances could result from possible engagement in higher risk sex (for example, commercial sex). A further examination (analysis not shown) suggests that women in female-headed households are more than twice as likely to report engaging in risky sex (last sex involved non-spousal partner without condom use) compared to their counterparts in male-headed households.

The observed higher risk of HIV infection among those with higher HIV/AIDS awareness may be as a result of those infected with HIV discovering they are HIV positive when they develop AIDS symptoms and becoming more aware about HIV/AIDS as a consequence. Also, the fact that the higher risk of infection among those with greater HIV/AIDS awareness is reduced when sexual behaviour factors are controlled for might suggest that those with a better awareness of modes of HIV transmission and how infection can be prevented are possibly adopting safer sexual behaviour to reduce their risk of infection. Although one might expect the association between HIV/AIDS awareness and the risk of infection to differ by gender, since previous studies have suggested that men have more control of risky behaviour (Hedden *et al.* 2009), implying that men with better awareness would be more likely to avoid risky behaviour than women, this article provides no evidence of a significant sex difference.

Interesting patterns of gender disparity are observed in relation to union/marital or sexual behaviour factors. Although previous studies had indicated that marriage was a risk factor of HIV infection among women (Glynn *et al.* 2001, World Health Organization (WHO) 2003), the evidence presented here suggests the contrary. In fact, marriage seems protective for HIV infection among women. In general, early marriage is associated with a reduced sex difference in HIV infection and the sex disparity is greater among those who are unmarried (that is, never married, widowed, divorced or separated) than among those married, especially those married in monogamous unions. Nevertheless, the observed patterns in the risk of HIV infection by union/marital status should be interpreted with caution, given the cross-sectional nature of the data analysed with no information on time sequencing of events of interest. It is possible that the current marital/union status may have been a consequence of HIV infection (that is, those who are HIV positive remaining single or being more likely to be separated/divorced or to have been widowed), rather than a risk factor. Therefore, the patterns observed here are mere associations since we cannot infer precise causal relationships, given the cross-sectional nature of data analysed.

Finally, the results with respect to sexual behaviour risk factors are as might be expected with earlier initiation of sexual activity, premarital sex, risky sexual behaviour (non-condom use with non-spousal partner) and multiple sex partners all being associated with an increased risk of HIV infection. The sex difference patterns suggest that sexual abstinence is more protective for women than men in reducing the risk of HIV infection, and that earlier sexual debut is more of a risk factor for women than men. The latter is consistent with patterns observed in previous studies suggesting that an earlier sexual debut was particularly risk-inducing for women (Boileau *et al*. 2009). It is possible that girls who initiate sexual activity at an early age have a particularly high risk of infection due to their increased biological susceptibility arising from possible tearing of sensitive tissues. A recent study on early sexual debut and associated HIV risk factors in South Africa observed that an early coital debut is associated with factors that may increase a young person’s risk of HIV infection, such as forced sex and having older partners (Pettifor *et al.* 2009). However, no significant differences were observed between men and women with respect to other sexual behaviour risk factors such as multiple sex partners, consistent with patterns observed in a systematic review of epidemiological studies in the sub-Saharan Africa region (Chen *et al.* 2007).

Overall, this article provides a general picture of the patterns in gender disparity in HIV infection across countries in sub-Saharan Africa. For the most part, the patterns observed are consistent with those observed in specific settings from previous studies. Also, the gender disparity varies only marginally across countries, suggesting that the patterns observed may be generalised across countries in the region. In addition, the analysis reveals interesting new perspectives, for instance, with respect to women living in female-headed households or not in stable union/marital partnerships that require further investigation.

## Footnotes

1 This has been attributable to women having a larger surface area of mucous membrane exposed during sexual intercourse and also because they are exposed to a larger quantity of infectious fluid (semen) than men.

2 Coerced sex or rape may lead to the tearing of sensitive tissues and increase the risk of contracting the HIV virus.

3 ‘The testing protocol undergoes a host country ethical review as well as an ethical review at ICF Macro. In countries with centres for disease control and prevention (CDC) involvement, the testing protocol is also reviewed by CDC. The testing is simple; in most cases the interviewer collects dried blood spots on filter paper from a finger prick and the filter paper is transported to a laboratory for testing. The laboratory protocol includes an initial enzyme-linked immunosorbent assay (ELISA) test, and then retesting all positive tests and 5–10% of the negative tests with a second ELISA. For tests with discordant results on the two ELISA tests, another test, usually a Western blot, is used to determine the result’ (ICF Macro 2010: 4).

4 The multilevel logistic regression analysis was undertaken using MLwiN software and estimations based on a second-order predictive quasi-likelihood procedure (Rasbash *et al.* 2009).

5 Since the country-level random effect has a standard normal distribution, the values are varied from −2 to +2, representing countries with the lowest and highest risk of HIV infection, respectively. We expect 95% of a standard normal distribution to lie between −1.96 and 1.96, hence, the values of ± 2 used here represent cut-off points for the extreme 5% at the tails of the distribution – that is, the 2.5% lowest and the 2.5% highest cases.

## Supporting Information

**Table A1.** Coverage for HIV testing by country and sex of respondent

**Table A2.** A description of variables used in the analysis

**Table A3.** DHS questions used to derive HIV/AIDS awareness index

## References

[b1] Ackermann L, De Klerk GW (2002). Social factors that make South African women vulnerable to HIV infection. Health Care for Woman International.

[b2] Babalola S, Tambashe BO, Vondrasek C (2005). Parental factors and sexual risk-taking among young people in Côte d’Ivoire. African Journal of Reproductive Health.

[b3] Boileau C, Zunzunegui MV, Rashed S (2009). Gender differences in unsafe sexual behavior among young people in urban Mali. AIDS Care – Psychological and Socio-Medical Aspects of AIDS/HIV.

[b4] Chen L, Jha P, Stirling B, Sgaier SK (2007). Sexual risk factors for HIV infection in early and advanced HIV epidemics in sub-Saharan Africa: systematic overview of 68 epidemiological studies. PLoS ONE.

[b5] Clark S, Bruce J, Dude A (2006). Protecting young women from HIV/AIDS: the case against child and adolescent marriage. International Family Planning Perspectives.

[b6] Dunkle KL, Jewkes RKBrown, H.C etal (2004). Gender-based violence, relationship power, and risk of HIV infection in women attending antenatal clinics in South Africa. Lancet.

[b7] Glynn JR, Caraël M, Auvert B, Kahindo M (2001). Why do young women have a much higher prevalence of HIV than young men?. A study in Kisumu, Kenya and Ndola, Zambia, AIDS.

[b8] Goldstein H (2003). Multilevel statistical models.

[b9] Gouws E, Stanecki K, Lyerla R, Ghys P (2008). The epidemiology of HIV infection among young people aged 15–24 years in southern Africa. AIDS.

[b10] Hedden SL, Whitaker D, Floyd L, Latimer WW (2009). Gender differences in the prevalence and behavioral risk factors of HIV in South African drug users. AIDS and Behavior.

[b11] ICF Macro (2010). HIV Prevalence Estimates from the Demographic and Health Surveys.

[b12] Jewkes RK, Levin JB, Penn-Kekana LA (2003). Gender inequalities, intimate partner violence and HIV preventive practices: findings of a South African cross-sectional study. Social Science and Medicine.

[b13] Langen TT (2005). Gender power imbalance on women’s capacity to negotiate self-protection against HIV/AIDS in Botswana and South Africa. African Health Sciences.

[b14] MacPhail C, Williams BG, Campbell C (2002). Relative risk of HIV infection among young men and women in a South African township. International Journal of STD and AIDS.

[b15] Madkan VK, Giancola AA, Sra KK, Tyring SK (2006). Sex differences in the transmission, prevention and disease manifestations of sexually transmitted diseases. Archives of Dermatology.

[b16] Ngom P, Magadi MA, Owuor T (2003). Parental presence and adolescent reproductive health among the Nairobi urban poor Journal of Adolescent Health.

[b17] Nicolosi A, Leite MLC, Musicco M, Arici C, Gavazzeni G, Lazzarin A (1994). The efficiency of male-to-female and female-to-male sexual transmission of the human immunodeficiency virus: a study of 730 stable couples. Epidemiology.

[b18] Obermeyer CM, Sankara A, Bastien V, Parsons M (2009). Gender and HIV testing in Burkina Faso: an exploratory study. Social Science and Medicine.

[b19] Pettifor A, O’Brien K, Macphail C, Miller WC, Rees H (2009). Early coital debut and associated HIV risk factors among young women and men in South Africa. International Perspectives on Sexual and Reproductive Health.

[b20] Rasbash J, Steele F, Browne W, Goldstein H (2009). A Users Guide to MLwiN, Version 2.10.

[b21] Rosenthal D, Smith A, Visser R (1999). Personal and social factors influencing age at first sexual intercourse. Archives of Sexual Behavior.

[b22] Sa Z, Larsen U (2008). Gender inequality increases women’s risk of HIV infection in Moshi. Tanzania, Journal of Biosocial Science.

[b23] Türmen T (2003). Gender and HIV/AIDS. International Journal of Gynecology and Obstetrics.

[b24] UNAIDS (2003). HIV/AIDS and Young People: Hope for Tomorrow.

[b25] UNAIDS (2004). Women and HIV/AIDS: Confronting the Crisis.

[b26] UNAIDS (2008). 2008 Global AIDS Epidemic Update.

[b27] UNAIDS and WHO (2009). AIDS Epidemic Update.

[b28] Upchurch DM, Kusunoki Y (2004). Associations between forced sex, sexual and protective practices, and sexually transmitted diseases among a national sample of adolescent girls. Women’s Health Issues.

[b29] World Health Organization (WHO) (2003). Gender and HIV/AIDS Gender and Health.

[b59] Van Der Straten A, King R, Grinstead O, Vittinghoff E (1998). Sexual coercion, physical violence, and HIV infection among women in steady relationships in Kigali. Rwanda, AIDS and Behavior.

