# Supplementary material for: Understanding the gender disparity in HIV infection across countries in sub-Saharan Africa: evidence from the Demographic and Health Surveys
Source: Sociol Health Illn. 2011 May 4;33(4):522–39. doi: 10.1111/j.1467-9566.2010.01304.x (PMC3412216; doi:10.1111/j.1467-9566.2010.01304.x)
Supplement: Supplementary file 1 [file shil0033-0522-SD1.pdf]

Table A1 coverage for HIV testing by country and sex of respondent

Country	HIV testing status							
	Women				Men			
	Tested	Refused	Other /missing	Cases	Tested	Refused	Other /missing	Cases
Burkina Faso 2003	91.7	3.5	4.8	4575	83.9	4.5	11.6	5984
Cameroon 2004	90.4	3.7	5.9	5703	88.9	3.7	7.4	5676
Cote d'Ivoire 2005	79.1	10.6	10.3	5772	76.3	11.1	12.6	5148
DR Congo 2007	90.3	4.4	5.3	5127	86.3	5.7	8.0	4 985
Ethiopia 2005	83.2	11.2	5.6	7142	75.4	12.6	12.0	6778
Ghana 2003	89.0	4.8	6.2	5949	79.8	9.7	10.5	5345
Guinea 2005	91.8	5.7	2.5	4189	87.2	5.0	7.8	5560
Kenya 2003	76.1	12.9	11.0	4303	69.7	10.5	19.8	4183
Lesotho 2004-05	80.4	10.7	8.9	3758	67.6	13.3	19.1	3305
Liberia 2007	87.0	7.3	5.7	7448	80.4	11.3	8.3	6476
Malawi 2004	70.4	22.5	7.1	4071	63.3	21.9	14.8	3797
Mali 2006	92.0	3.2	4.8	5157	83.7	4.8	11.5	4643
Niger 2006	87.8	4.0	8.2	8738	84.2	4.7	11.1	3839
Rwanda 2005	97.0	0.9	2.1	5837	95.3	1.6	3.1	4959
Senegal 2005	84.5	9.9	5.6	5350	75.5	16.0	8.5	4375
Sierra Leone 2008	87.7	6.3	3.3	3954	85.0	7.5	4.8	3541
Swaziland 2006	87.2	9.5	3.3	5301	77.6	16.6	5.8	4675
Tanzania 2003/4	83.5	12.3	4.2	7154	77.0	13.9	9.1	6196
Zambia 2007	77.1	19.9	3.0	7408	72.2	20.1	7.7	7146
Zimbabwe 2005/06	75.9	13.2	10.9	9870	63.4	17.4	19.2	8761

Source: Compiled from each country's DHS report.

Table A2 A description of variables used in the analysis

Variable	Description[1]
Response variable	
HIV status	Based on HIV test result (HIV03) from the HIV data file, coded as 1 for those who tested HIV positive, and 0 otherwise.
Explanatory variables	
Gender (main study variable)	Cases from the women sample are assigned a code of 1, while men are assigned 0 and used as the reference category.
Age group	The DHS obtains data on age in completed years in the individual interview which is used to derive 5-year age groups (V013). This has been re-classified into broader age groups (15-19, 20-29, 30-39, 40+), with the last age group (40+) used as the reference category.
Residence	The DHS variable ‘type of place of residence’ (V025) is coded as 1 for ‘rural’ and 0 for ‘urban’ used as the reference category.
Education level	Information on the ‘highest educational level’ (V106) is reclassified into: 0-none; 1-primary; and 2- secondary or higher, with ‘none’ used as the reference category.
Sex of household head	Information on ‘sex of household head’ from the household schedule (V151) is coded as 1 for female and 0 for male (reference category)
Wealth quintile	Based on the variable ‘wealth index’ (V190), derived by the DHS through Principal Components approach, using information on household possessions and amenities from the household schedule. The ‘poorest/lowest’ is used as reference category.
Religion	Information on religion (V130) obtained from the individual interview is coded as 1 – Catholic /Orthodox (ref.), 2-protestant/other Christian, and 3-Muslim/other.
HIV/AIDS awareness	A summary measure derived from questions on knowledge of modes of HIV transmission and ways of avoiding infection (see Table A3), classified into 1-low (ref.), 2-average and 3-high.
Marital status	Information on current marital status (V501) and number of other wives (V505) used to derive marital status which is classified into 1-never married, 2-married/living together-monogamous (ref.), 3-married/living together – polygamous, and 3-divorced/separated/widowed.
Age at first marriage	Information on age at first marriage (V511) is coded as 1-‘never married’ 2-‘<16’, 3-’16-17’, 4-’18-19’, and 5-’20 or older’ (ref.).
Age at first sex	Information on age at first sex (V525) is coded as 1-‘never had sex’ 2-‘<16’, 3-’16-17’, 4-’18-19’, and 5-’20 or older’ (ref.). For those who had first had sex when they first married, age at first sex equals age at first marriage.
Premarital sex	Variable for whether or not the responded had sex before marriage, coded as 1 if age at first sex is less than age at first marriage, or respondent ever had sex but has never married, and coded as 0 otherwise (ref.)
Risky sexual behaviour	Coded as 1 if respondent did not use condoms during last sex (V761) with a non-spousal partner (V767a), and coded as 0 otherwise (ref.).
Multiple sex partners	Coded as 1 if respondent reported more than one sex partner within the 12 months preceding the survey (V766B), and coded as 0 otherwise (ref.)

[1] The variable names shown are for the women sample, which are similar to variable names for the men sample, but with letter ‘M’ added at the beginning (e.g. V013 for women is equivalence to MV013 for men).

Table A3 DHS questions used to derive HIV/AIDS awareness index

Statement	Yes	No
Ever heard of AIDS	1	0
Reduce chance of HIV/AIDS by using condoms	1	0
Reduce chance of HIV/AIDS by having only one sexual partner	1	0
HIV/AIDS can be transmitted through mosquito bites	0	1
HIV/AIDS can be transmitted by sharing utensils	0	1
A health looking person can have AIDS virus	1	0
HIV/AIDS can be transmitted through pregnancy	1	0
HIV/AIDS can be transmitted through breastfeeding	1	0
